# A recalcitrant case of pemphigus treated with inebilizumab

**DOI:** 10.1016/j.jdcr.2025.08.042

**Published:** 2025-10-08

**Authors:** Christina M. Bear, Amir Reza Djavid, Braden Candela

**Affiliations:** Department of Dermatology, Columbia University Irving Medical Center, New York, New York

**Keywords:** desmoglein, Dsg1, Dsg3, immunobiologics, immunosuppressives, inebilizumab, monoclonal antibody, mucosal, oral, paraneoplastic pemphigus, pemphigus, pemphigus vulgaris, prednisone, recalcitrant, stomatitis

## Introduction

Pemphigus is a B-cell–mediated autoimmune blistering disease. Autoantibodies target desmosomal proteins, such as desmoglein (Dsg) 1 and 3, disrupting keratinocyte adhesion in the skin and mucosa and leading to blistering and painful erosions, often with hemorrhagic crusts. One standard immunosuppressive therapy is rituximab, an anti-CD20 antibody that depletes most B cells, including those reactive to desmosomal antigens.

In this case report, elevated anti-Dsg1/Dsg3 levels and positive monkey esophagus indirect immunofluorescence supported a diagnosis of pemphigus vulgaris, while a history of thymoma raised concern for paraneoplastic pemphigus (PNP). To reflect this overlap, we refer to this patient’s condition as “pemphigus”. The patient’s mucocutaneous pemphigus was resistant to rituximab and other immunosuppressants. Given the role of B-cell autoantibodies in pemphigus, treatment was initiated with inebilizumab (INE), resulting in significant improvement. This report suggests INE may be an effective treatment option for recalcitrant pemphigus.

## Case report

A 72-year-old Eastern European man with a history of bilateral renal oncocytoma presented with 2 years of widespread, painful, erosive plaques on the torso and extremities, severe stomatitis, and oral pain. Systemic workup identified a thymoma; after resection, there was no further evidence of disease on positron emission tomography–computed tomography or magnetic resonance imaging and no indication for systemic therapy. Despite this, the patient’s mucositis, oral pain, and skin lesions persisted, raising concern for PNP and prompting referral to our dermatology center.

**Fig 1:**
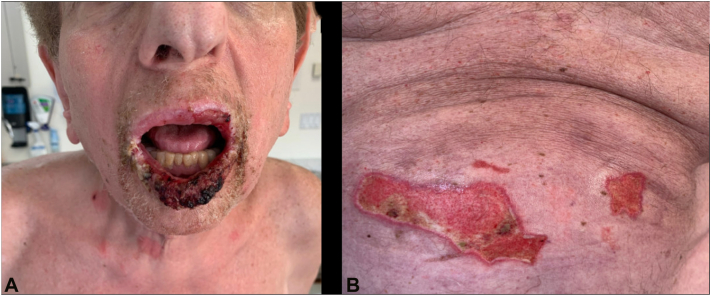
**A,** Clinical findings at initial visit. Severe oral stomatitis. Dsg1: 163 and Dsg3: 66; **(B)** Clinical findings at initial visit. Extensive crusted erosions on torso and extremities. Dsg1: 163 and Dsg3: 66.

Initial immunobullous workup included Dsg1 levels of 163, Dsg3 levels of 66, negative envoplakin, and indirect immunofluorescence serum findings with positive monkey esophagus and negative rat bladder reactivity. These serologic findings supported a diagnosis of “pemphigus”, highlighted by clinical and diagnostic overlap between pemphigus vulgaris and PNP. Soon after, the patient was initiated on a 6-dose rituximab regimen over 4 months. During this time, he was also started on prednisone 70 mg oral daily, 10 mg oral tofacitinib twice daily, and weekly intravenous immunoglobulin (IVIG) as a steroid-sparing agent (Fig 1, *A* and *B*).

**Fig 2:**
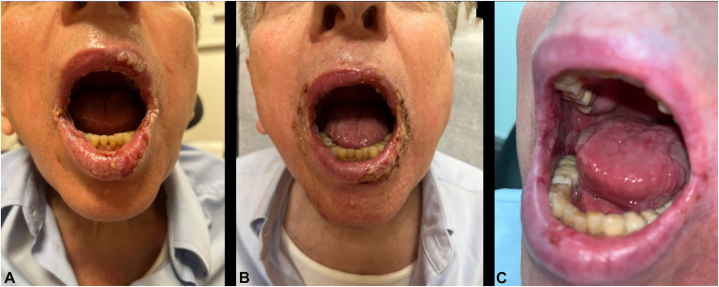
**A,** Oral mucosa immediately following rituximab 6-dose regimen, with improvement of mucosal blisters with decreased crusting and hemorrhage. Dsg1: 132 and Dsg3: 43; **(B)** 4 months following rituximab, showing worsened lip and intraoral mucosal stomatitis, demonstrating recalcitrant disease. Dsg1: 75 and Dsg3: 14; and **(C)** 1 month following completion of inebilizumab treatment. Dsg1: 23 and Dsg3: <9.

After several months with minimal improvement, tofacitinib was transitioned to mycophenolate. One month after completing rituximab, oral mucosa showed slow improvement, and several back erosions resolved with postinflammatory hyperpigmentation. Although B cell levels were depleted, Dsg1 and Dsg3 levels remained elevated at 132 and 43, respectively (Fig 2, *A*). New blisters soon developed on the trunk and extremities.

Several months later, bloodwork revealed decreasing Dsg1: 95 to 107 and Dsg3: 28 to 48. IVIG was redosed, prednisone tapered to 60 mg daily, and Dsg levels continued to decrease. Mycophenolate was discontinued due to lymphopenia (Fig 2, *B*).

Due to persistent disease after multiple first-line treatments, alternative treatment was considered. INE, an anti-CD19 monoclonal antibody, emerged as a promising option given its efficacy in another B-cell–mediated autoimmune disorder, neuromyelitis optica (NMO). NMO is a rare inflammatory disorder involving autoantibodies to Aquaporin-4, a transmembrane water channel in astrocyte foot processes, resulting in numerous neurologic sequelae.^1^ In NMO, INE targets immunoglobulin G–producing cells, reducing circulation of pathologic anti-Aquaporin 4 antibodies. Our group theorized INE could be used in pemphigus given its similar pathogenic process to NMO, to help eliminate recalcitrant circulating pemphigus autoantibodies.

Given this clinical reasoning, the patient received 2 300 mg INE infusions, 2 weeks apart, to deplete pathogenic antibody-producing B cells. He displayed significant improvement, with near resolution of lip crusting and back/extremity erosions, with only mild residual oral mucosal disease. One month after the second INE dose, he presented with a left arm abscess that cultured positive for *Mycobacterium chelonae* and was treated with azithromycin and trimethoprim/sulfamethoxazole. Subsequently, the prednisone was tapered from 30 mg to 5 mg daily. Four months after his final INE infusion, Dsg levels were Dsg1: 23 and Dsg3: <9. He has since remained stable without flares (Fig 2, *C*).

## Discussion

In this case, the patient’s mucocutaneous pemphigus was resistant to rituximab and other immunosuppressants, including mycophenolate mofetil and tofacitinib. Despite a combination of high-dose prednisone, rituximab, and IVIG, Dsg1 and Dsg3 levels remained elevated, and symptoms only improved slightly. He continued developing new erosions, with worsening crusts and severe oral pain that limited eating and drinking.

The decision to trial INE was informed by data suggesting that despite rituximab B cell depletion, some pemphigus patients experience reconstitution of CD19+ anti-Dsg autoreactive memory B cells, leading to relapse or recalcitrant disease.^2^ With the primary goal of depleting B cells to treat pemphigus, rituximab targets only CD20-expressing B cells, leaving anti-Dsg plasma cells and plasmablasts in lymphoid tissues unaffected.^3^

**Fig 3:**
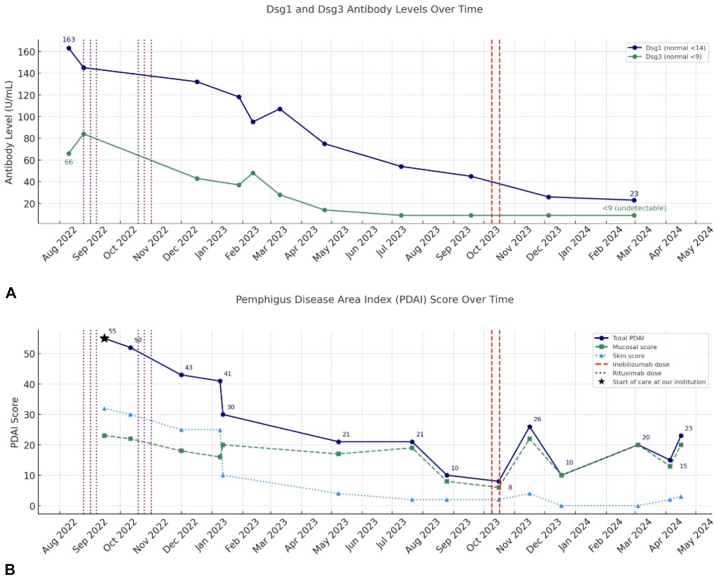
**A** and **B,** Antidesmoglein antibody levels and Pemphigus Disease Area Index (PDAI) scores in relation to time and therapeutic regimen.

Studies suggest targeting CD19+ B cells could offer stronger depletion of anti-Dsg B cells. Halliley et al found CD19 is expressed on nearly all plasma and memory cells in secondary lymphoid organs and about half in bone marrow.^4^ INE, an anti-CD19 monoclonal antibody, targets circulating B cells and extensively penetrates the spleen and bone marrow, eliminating autoreactive B cells that could lead to disease relapse.^5^^,^^6^ Musette et al suggested anti-CD19 antibodies could treat pemphigus more aggressively, consistent with how INE has been used to more thoroughly deplete B cells in NMO.^7^^,^^8^ In this case, recalcitrant disease appeared to indicate incomplete depletion of B cells by first-line treatments, making INE a rational choice for targeting all B-lineage cells, including plasma, plasmablast, and memory cells in blood and lymphoid tissues. Known INE side effects include increased infection susceptibility, headache, drowsiness, fever, rash, nausea, or muscle aches. While not currently a standard pemphigus therapy, INE effectively reduced Dsg1 and Dsg3 levels to normal, resolved symptoms, reduced Pemphigus Disease Activity Index scores, and allowed prednisone tapering to 5 mg per day (Fig 3, *A* and *B*).

Several considerations come with using INE for recalcitrant pemphigus. The primary concern is increased infection susceptibility. This patient developed cutaneous *Mycobacterium chelonae*, likely due to T-cell depletion from prednisone combined with other immunosuppressants, including mycophenolate. Aside from this, the patient did not experience other notable side effects. Confounding factors, such as fluctuating prednisone doses and the potentially delayed effects of rituximab (which can take 15 days to 3 months), should also be considered.^9^

This case demonstrates the potential use of INE as an effective treatment option for pemphigus resistant to typical first-line immunosuppressive medications.

## Conflicts of interest

None disclosed.
